# Vasoactive drugs and the distribution of crystalloid fluid during acute sepsis

**DOI:** 10.1016/j.jointm.2025.08.008

**Published:** 2025-10-17

**Authors:** Robert G. Hahn, Yuhong Li, Randal O. Dull

**Affiliations:** 1 Department of Clinical Sciences at Danderyd Hospital, Karolinska Institutet, Stockholm, Sweden; 2 Department of Anesthesiology, Shulan (Hangzhou) Hospital, Shulan International Medical College, Zhejiang Shuren University, Hangzhou, China; 3 Department of Anesthesiology, Department of Pathology, and the Department of Surgery, University of Arizona College of Medicine, Tucson, AZ, USA

**Keywords:** Animal model, Adrenergic receptors, Fluid therapy, Norepinephrine, Pharmacokinetics, Phenylephrine

## Abstract

**Background:**

Crystalloid fluid is combined with vasoactive drugs when treating acute sepsis. We used kinetic analysis to compare the effects of vasoactive drugs on the hemodynamics and the volume kinetics of crystalloid fluid in non-septic and septic sheep. Specific attention was given to the effects of drugs and sepsis on fluid distribution to the “third fluid space.”

**Methods:**

Forty-nine sheep were studied, of which 25 were made septic by cecal puncture and administration of lipopolysaccharide. A bolus infusion of 20–25 mL/kg of crystalloid fluid was given 1 h later, together with therapeutic doses of phenylephrine, norepinephrine, isoprenaline, dopamine, or esmolol. Central hemodynamics were monitored, and blood samples for the calculation of volume kinetics were taken. The distribution and elimination of the infused volume were calculated using mixed-model kinetics with extensive covariate analyses based on 1134 measurements of the hemoglobin-derived plasma dilution (23 per experiment) and 193 measurements of urine output collected over 180 min.

**Results:**

Sepsis induced a hyperkinetic, hypotensive, and vasodilatory state. Cardiac output averaged 12.1 L/min in septic sheep *vs*. 7.6 L/min in non-septic sheep, and mean arterial pressure was 72 *vs*. 109 mmHg, respectively. Differences in hemodynamic variables between the non-septic and septic sheep were all statistically significant (*P* <0.001). However, the modifying influences of vasopressors on hemodynamics and fluid distribution were weakened in the septic state. The rate constant for urine output (*k*_10_) was strongly decreased by sepsis, modestly reduced by β_1_-adrenergic stimulation, and increased by α_1_-adrenergic stimulation. Fluid was able to access the remote slow-exchange interstitial fluid space (“third fluid space”) only during sepsis. In theory, fluid accumulation in this space would decrease by α_1_-adrenergic stimulation and increase by β_1_-adrenergic stimulation, but differences were small due to the overall weak effects of adrenergic drugs in the septic sheep. We hypothesize that the changeover from brisk urine flow in the non-septic sheep to marked accumulation of fluid in the “third fluid space” was due to inflammation-induced alterations in the interstitial matrix.

**Conclusion:**

Vasoactive drugs were less effective in septic compared to non-septic sheep. Most importantly, acute sepsis was characterized by a marked accumulation of fluid in an interstitial fluid space with slow turnover.

## Introduction

Sepsis continues to be a major cause of morbidity and mortality in both the post-surgical period and medical intensive care units.^[^^1^^,^^2^^]^ The derangements in cardiovascular performance are complex and often require both fluid boluses and vasopressors to normalize circulatory parameters.^[^[3], [4], [5]^]^ Part of the cardiovascular dysfunction is related to alterations in the distribution of fluid between the vascular compartment and the interstitial space.^[^^6^^]^ These effects are of great importance, as maldistribution of fluid may progress to septic shock. Large volume resuscitation, commonly administered as part of sepsis protocols, then leads to marked peripheral edema that can contribute to organ dysfunction.

Administration of vasopressors in a sheep model has demonstrated different effects on fluid distribution and plasma volume (PV) expansion, depending on the adrenergic receptors that were activated.^[^^7^^]^ The combined effect of vasopressors and fluid resuscitation on septic physiology is, however, unknown but has implications in developing pharmacological strategies to correct PV and urine output, while minimizing tissue edema. We have developed a line of investigation centered on the interstitial space and sub-compartments that influence the movement of fluid and, specifically, the return of fluid to the central circulation via transcapillary reabsorption and the lymphatic system.^[^^8^^,^^9^^]^ The kinetics of the interstitial fluid and, particularly, of the “third fluid space,” may have significant implications in the management of sepsis.

We studied these questions by applying population (log-likelihood) mathematics to fluid volume kinetic data from two studies of adrenergic drugs in sheep that were performed in a similar way. One study was undertaken in healthy sheep^[^^7^^]^ and the second in septic sheep.^[^^10^^]^ The hypothesis was that sepsis alters the hemodynamics and the distribution of crystalloid fluid in a way that can be deliberately modified by adrenergic stimulation. Special attention was given to the role of the “third fluid space” in sepsis.

## Methods

This study is a secondary analysis of two series of experiments: one performed in non-septic and the other in acutely septic sheep. The hemodynamics were monitored and the influence of adrenergic drugs on the distribution and elimination of infused crystalloid fluid was quantified.

### Non-septic sheep

The non-septic study consisted of 24 experiments. Six adult healthy sheep weighing 29–35 kg (mean 32 kg) each underwent four experiments during which isotonic (0.9%) saline, 24 mL/kg, was infused over 20 min. An adrenergic drug was given by a constant rate intravenous (i.v.) infusion during three of the experiments, while no drug was given during the fourth infusion experiment. The drug infusions were continued for 3 h. Animals were awake.

### Septic sheep

The study of acute sepsis included 25 healthy adult sheep weighing 14–26 kg (mean, 20 kg) that each underwent only one experiment. The sheep were under general anesthesia when sepsis was created by cecal ligation and puncture, combined with a 10-min i.v. infusion of lipopolysaccharide (0.5 mg/kg).^[^^11^^,^^12^^]^ The animals were then randomly allocated to receive no drug or one of 4 adrenergic drugs at a constant rate i.v. infusion over 2.5 h, beginning 50 min after the sepsis induction. After an additional 10 min, an i.v. infusion of Ringer’s lactate (20 mL/kg) over 30 min was initiated. All sheep survived the experiment but were sacrificed at the end of the study.

### Adrenergic drugs

We investigated how stimulation of three adrenergic receptors (α_1_-, β_1_-, or dopamine) influenced the fluid kinetics. For this purpose, 3 µg/( kg·min) of phenylephrine and 50 µg/ (kg·min) of dopamine (both studies) were administered. Moreover, one group in the non-septic sheep study was given isoprenaline (0.1 µg/ (kg·min)) and, in the septic sheep, norepinephrine (1 µg/ kg·min)) or esmolol (β_1_-receptor blocker; 50 µg/ (kg· min))^[^^13^^]^ was given. The receptors and the drugs used for stimulation are listed in Table 1.

**Table 1:** The level of stimulation of receptors used in the volume kinetic analysis.

Drug treatment	α_1_-Adrenergic receptors	β_1_-Adrenergic receptors	Dopamine receptors	Dose (µg/ (kg· min))	Data points (*n*)
None (control)	0	0	0	0	260
Phenylephrine	3	0	0	3	250
Isoprenaline	0	3	0	0.1	150
Norepinephrine	3	2	0	1	105
Dopamine	0	1	2	50	259
Esmolol	0	–1	0	50	109

Zero means no effect, +3 strong effect, and −1 means inhibition.

### Measurements

Water was withdrawn 24 h before the experiments. Invasive hemodynamic measurements were performed in both series.

The non-septic sheep used a femoral and pulmonary artery catheter to measure cardiac output (CO), mean arterial pressure (MAP), and the central venous pressure (CVP). Data were displayed on a Hewlett-Packard Monitor model 78901 A (Hewlett-Packard, Andover, MA, USA).

In the septic sheep, the femoral artery and one internal jugular vein were cannulated for measurement of CO, MAP, and CVP using the FloTrac/Vigileo system (Edwards Lifesciences, Irvine, *CA*, USA). Here, the CO data were calibrated against thermodilution.

In both series of experiments, systemic vascular resistance (SVR) was calculated as ([MAP–CVP] 80/CO). Arterial blood samples (1–2 mL) were drawn for hemoglobin (Hb) measurement using a 482 CO—Oximeter or a GEM Premier 3000 (Instrumentation Laboratory System, Lexington, MA, USA) on 23 occasions during each experiment. The urine output was collected and quantified via a catheter placed in the bladder.

### Kinetic model

Volume kinetic analysis is a method for quantification of fluid volume shifts between body fluid compartments.^[^^14^^]^ The method has similarities to drug pharmacokinetics but uses the dilution of non-diffusible components of the blood, such as Hb, to serve as an index of PV expansion. The analysis uses rate constants to identify short delays in the distribution of fluid between functional compartments that can be interpreted in quasi-physiological terms. An underlying assumption is that the flow from one space to another is the product of the volume expansion of that space and an associated rate constant. Receptor effects are identified by a deviation of a rate constant in one group from the values in the other groups.

A three-volume kinetic “base” model with five rate constants (*k*_12_, *k*_21_, *k*_10_, *k*_23_, and *k*_32_) and one scaling factor between dilution and volume (*V*_c_, central volume) was fitted to the frequently measured plasma dilution and the urinary excretion.

**Figure 1:**
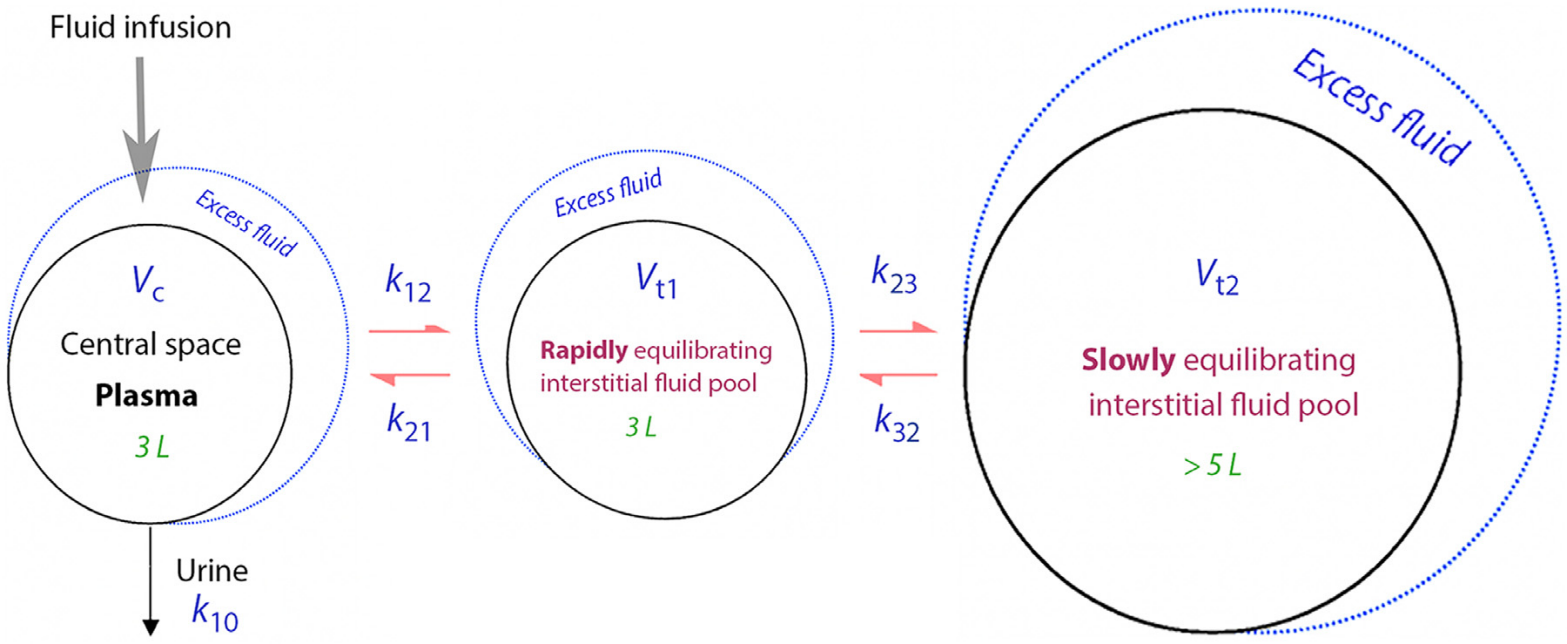
The kinetic model used for the analysis of fluid distribution. *k*_12_, *k*_21_, *k*_10_, *k*_23_, and *k*_32_: Rate constant; *V*_c_ : Central volume; *V*_t1_: extravascular space; *V*_t2_: third fluid space.

The kinetic model is illustrated in Figure 1 and is intended to mimic human physiology. Briefly, fluid is infused into the plasma (*V*_c_), from which distribution occurs to an extravascular space (*V*_t1_), and further to a more remote fluid space (*V*_t2_, the “third fluid space”). Redistribution of the fluid occurs in the reverse order. Elimination is governed by the rate constant *k*_10_, which is obtained as the measured urine output divided by the modeled volume expansion of *V*_c_ during the same time period as the urine was collected.

The Hb-derived fractional plasma dilution was used to indicate the volume expansion of *V*_c_ resulting from the infusion, according to the following equation:

$$\mathrm{Plasma}\;\mathrm{dilution}=\frac{\left(Hb_{\mathrm{baseline}}/Hb_{\mathrm{later}}\right)-1}{1-{\mathrm{hematocrit}}_{\mathrm{baseline}}}$$

Each dilution underwent a minimal correction to account for blood sampling.^[^^15^^]^

### Kinetic calculations

The kinetic model was simultaneously fit to all measurements of plasma dilution and urinary excretion (dependent variables) using Phoenix software version 8.3.4 for nonlinear mixed effects (Pharsight, St. Louis, MO) with the First-Order Conditional Estimation Extended Least Squares (FOCE ELS) as the search routine.

Differences in fluid distribution caused by the adrenergic drugs were evaluated by covariance analysis based on the activity code for the receptor effect of each drug (α_1_, β_1_, or dopamine) according to the scheme shown Table 1.

Identification of appropriate covariates was guided by plots of random effects (“eta:s”) using both forward addition and backward removal of the most promising candidates.^[^^16^^]^ The criterion for accepting a covariate was that its inclusion should reduce the −2 LL (log likelihood) for the model by >3.84 points (*P* < 0.05) or >6.6 points (*P* < 0.01). In addition, the 95% confidence interval (CI) for the estimate was not allowed to include 0.

The goodness-of-fit and performance of the final model were evaluated using predictive checks, residual plots, and plots of the conditional weighted residuals (CWRES).

The differential equations for the kinetic model, the mathematical correction of plasma dilution for blood sampling, and the covariance models are explained in Supplementary File 1.

### Statistical analysis

Data showing a normal distribution are reported as the mean ± standard deviation (SD). Differences in hemodynamic variables between the non-septic and septic sheep were studied by one-way analysis of variance . Kinetic parameters are reported as the best estimate and 95% CI, according to the output from the Phoenix program. The significance levels for inclusion of the covariates were taken from the Phoenix program.

## Results

### Hemodynamics

CO was higher in the septic sheep (mean 12.1 *vs*. 7.6 L/min) while MAP was lower (72 *vs*. 109 mmHg) than in the non-septic sheep. The CVP (5.6 *vs*. 7.6 mmHg) and SVR (1408 *vs*. 481 dynes·s/cm^−5^) were also lower in the septic sheep, as was the ratio between urine output and the infused fluid volume (9% *vs*. 122%, respectively; all differences *P* < 0.001).

Vasoactive drugs markedly changed the vascular resistance in the healthy sheep, while changes were small in the septic sheep (Table 2).

**Table 2:** Hemodynamic measurements based on all data points and the ratio of urine output to infused fluid volume.

Measurements	Group	Control	Phenylephrine	Dopamine	Isoprenaline	Norepinephrine	Esmolol
Cardiac output (L/min)	Healthy Septic	5.2 ± 1.0 11.5 ± 4.2	4.3 ± 1.0 12.7 ± 4.6	10.2 ± 2.3 13.2 ± 5.3	11.4 ± 2.6 -	- 13.7 ± 4.1	- 10.1 ± 14.3
MAP (mmHg)	Healthy Septic	92 ± 7 66 ± 18	149 ± 15 75 ± 18	113 ± 20 76 ± 18	83 ± 7 -	- 74 ± 14	- 63 ± 13
CVP (mmHg)	Healthy Septic	7.8 ± 4.5 4.0 ± 3.6	8.0 ± 4.1 8.0 ± 3.9	8.4 ± 2.7 3.4 ± 3.7	5.1 ± 1.9 -	- 5.5 ± 2.3	- 6.4 ± 3.2
SVR (dynes·s/cm^−5^)	Healthy Septic	1355 ± 250 438 ± 151	2838 ± 303 472 ± 261	861 ± 255 536 ± 284	575 ± 140	452 ± 202	518 ± 199
Ratio of urine output/infused	Healthy Septic	1.18 (1.06–1.31) 0.04 (0.02–0.13)	2.00 (1.68–2.32) 0.15 (0.09–0.17)	1.61 (1.22–2.04) 0.04 (0.03–0.08)	0.21 (0.20–0.22) -	- 0.12 (0.05–0.17)	- 0.038 (0.01–0.07)

Data are presented as mean ± SD or median (interquartile range)

CVP: Central venous pressure; MAP: Mean arterial pressure; SVR: Systemic vascular resistance.

### Kinetic analysis

The kinetic analysis was based on 1134 measurements of plasma dilution and 193 of urine output. MAP and CVP were obtained on the same occasions as the measurements of plasma dilution, and CO on 87% of them. The Akaike criterion test confirmed that the three-volume model was statistically superior to the two-volume model.

**Figure 2:**
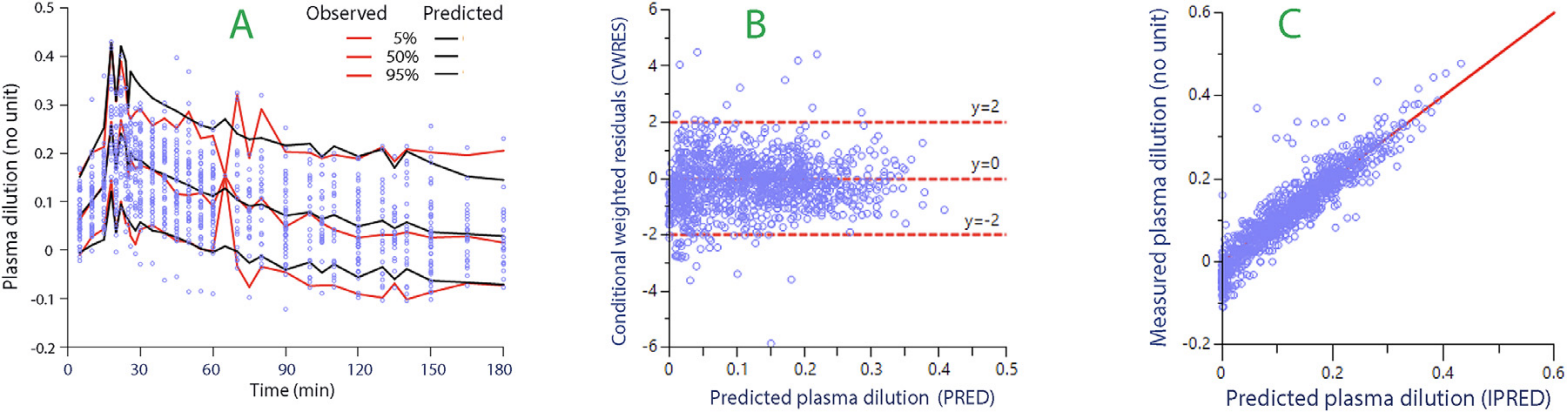
Goodness-of-fit diagnostics and predictive checks of the kinetic model. A: Predictive check. The observed confidence intervals (red lines) and the ones determined by 500 simulations using the parameters in the final model (black lines). Close agreement indicates good model specification. B: The conditional weighted residuals for the plasma dilution when predicted without consideration of covariates. Each point is one measurement. C: The relationship between the measured plasma dilution and the model-predicted plasma dilution when the covariates are considered.

The three-volume kinetic model was simultaneously fitted to all data on plasma dilution and urine output (Figure 1). The final parameter estimates are shown in Table 3, and performance measures for the model are shown in Figure 2.

**Table 3:** Population kinetic parameters for infused fluid volume in the final model.

Kinetic parameter	Covariate	Covariate model	Best estimate	95% CI	CV (%)	−2 LL
tv*V*_c_ (L)			1.22	1.10 to 1.35	5.3	
tv*k*_12_ (10^−3^/min)			56.7	47.4 to 66.0	8.4	
tv*k*_21_ (10^−3^/min)			63.5	54.4 to 72.5	7.2	
tv*k*_23_ (10^−5^/min)			4.47	4.09 to 4.86	4.4	
tv*k*_32_ (10^−5^/min)			86.9	79.6 to 94.2	4.3	
tv*k*_10_ (10^−3^/min)			47.4	39.9 to 54.9	8.1	−1133
*k*_12_	β_1_-receptor	Linear	62.8	57.6 to 68.0	4.2	−1161
*k*_21_	β_1_-receptor	Linear	−0.16	−0.18 to −0.14	−7.2	−1168
*k*_23_	During infusion	Exponential	−6.40	−7.07 to −5.72	−5.3	−1211
	Sepsis	Exponential	7.43	6.96 to 7.91	3.2	−1157
*k*_10_*k*_10_	β_1_-receptor	Linear	−0.40	−0.41 to −0.39	−1.8	−1190
	α_1_-receptor	Linear	0.45	0.38 to 0.51	7.6	−1201
	Sepsis	Exponential	−2.94	−3.37 to −2.52	−7.3	−1144
Full block model	All the above					−1241

Shown are the typical values (tv) for the fixed parameters in the group, followed by individual-specific covariates.

CI: Confidence interval; CV: Coefficient of variation (inter-individual); LL: Log likelihood for the model; tv: Typical value for the group; Vc: Central volume.

The mean adrenergic scores were: β_1_-receptor = 0.62; α_1_-receptor = 0.94.

Decrease by >3.84 = *P* < 0.05 and >6.6 = *P* < 0.01.

The complete equations for the parameters are shown in Supplementary File 1.

Acute sepsis increased the rate constant that governed flow into the “third fluid space” (*k*_23_) and strongly reduced the rate constant for urine flow (*k*_10_). Other kinetic differences between the groups were attributable to adrenergic receptor stimulation.

**Figure 3:**
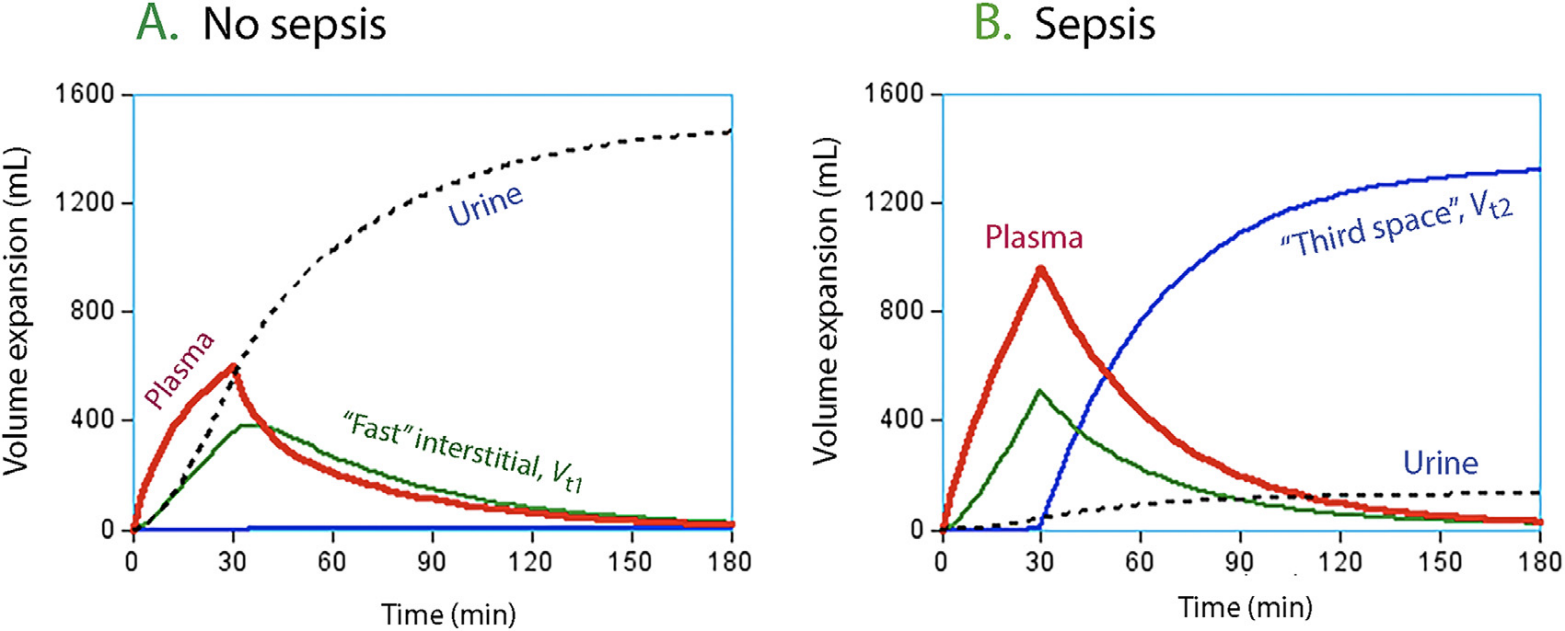
Stimulated distribution of isotonic saline in healthy and septic sheep. Simulation of the distribution of 1.5 L of isotonic saline infused over 30 min in healthy awake sheep (A) and septic anesthetized sheep (B). No effects of adrenergic receptor stimulation are included. Note that in healthy sheep, there is no fluid distribution to the *V*_t2_, the third space. Conversely, during sepsis, *V*_t2_ is the primary compartment for fluid accumulation.

**Figure 4:**
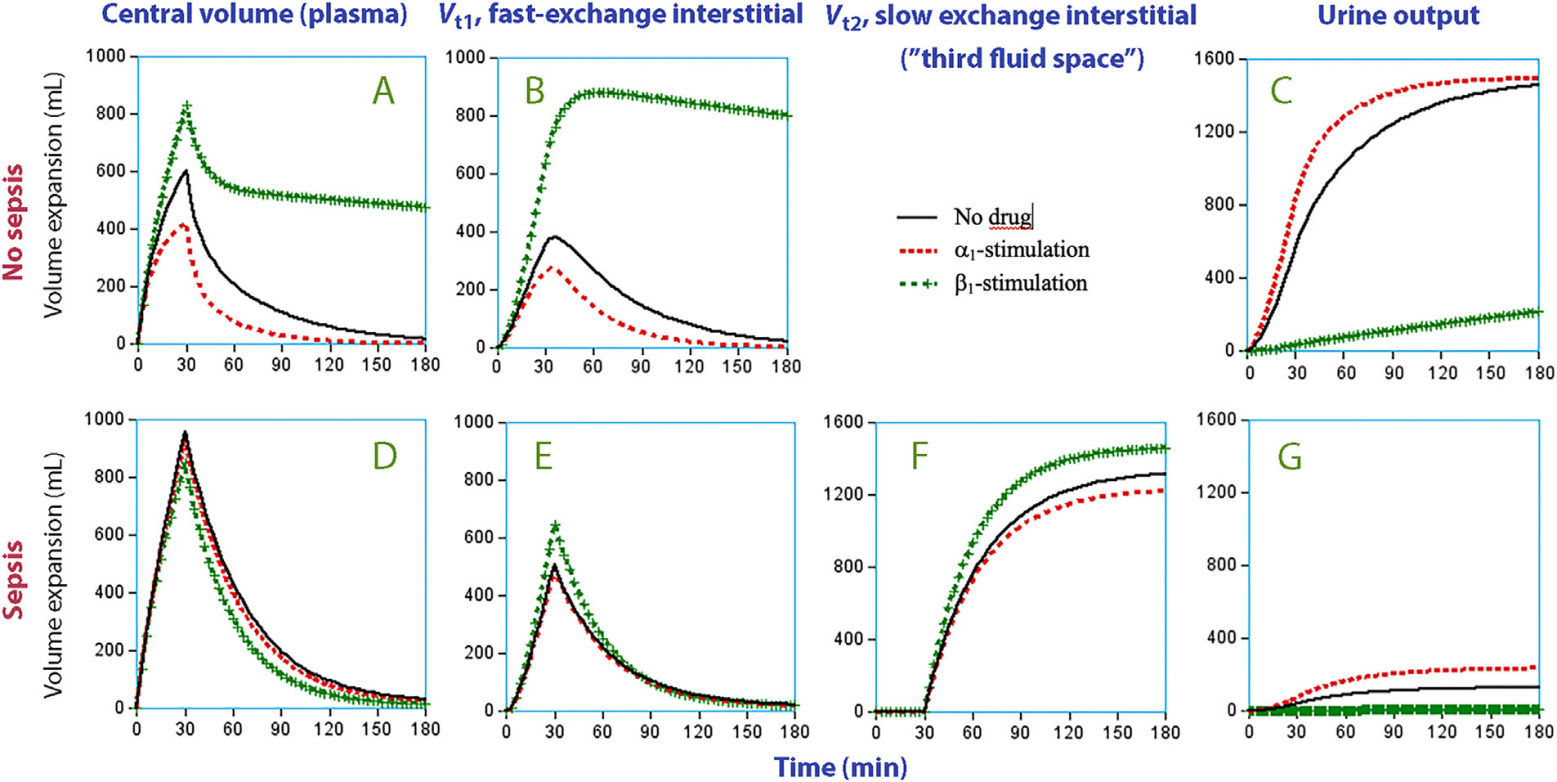
Simulated distribution of isotonic saline with strong adrenergic stimulation in healthy and septic sheep. Simulation of the distribution of 1.5 L of isotonic saline infused over 30 min in healthy awake sheep (A–C) and septic anesthetized sheep (D–G) . The effect of 3+ (strong) adrenergic stimulation is shown (see Table 1 for a list of adrenergic strengths). Note the scale difference between subplots C, F, and G *vs.* the others.

The central volume (e.g., PV) averaged 1.22 L at baseline (47 mL/kg, corresponding to a blood volume of 71 mL/kg). The rate constant for fluid entry to the “third space” (*k*_23_) was almost zero in the healthy sheep but greatly increased during sepsis. However, fluid cannot enter *V*_t2_ before *V*_t1_ is filled, and, therefore, the modeled PV expansion in response to fluid was initially greater in the septic sheep due to the consistently lower *k*_10_ (Figure 3). The combined effects of sepsis and the adrenergic modulators are illustrated in Figure 4.

### Adrenergic receptors

**Figure 5:**
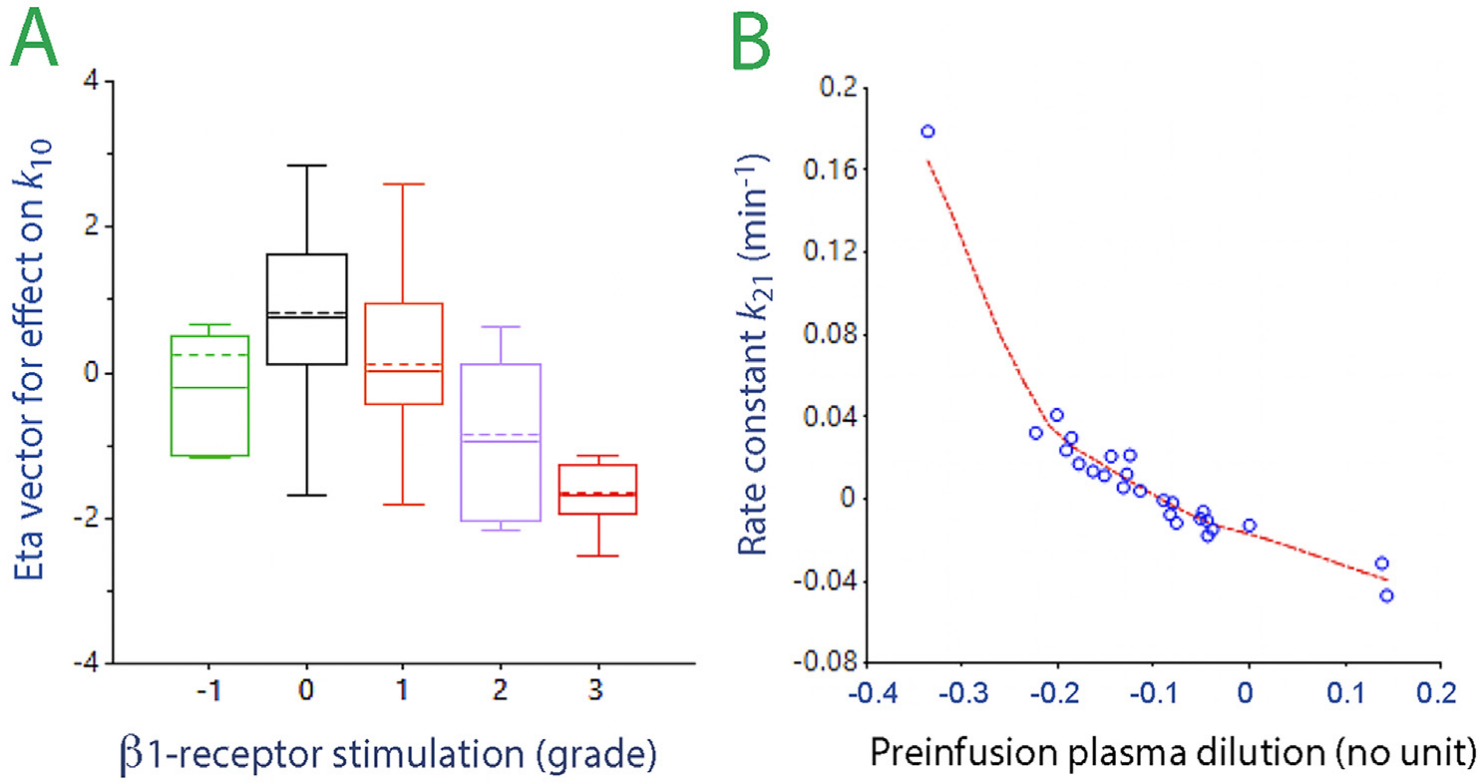
Covariates relationships in the kinetic model. A: The “eta vector” for the relationship between gradually more intense β_1_-adrenergic stimulation (for grades, see Table 1) and its inhibitory effect on *k*_10_, which governs the urine flow. The final kinetic analysis implemented this relationship as a linear function. B: The rate constant *k*_21_ during saline infusion *vs.* the sepsis-induced change in PV during 50 min before any fluid was given. Analysis of the septic sheep only. Each point is one experiment. Dilution of −0.1 implies a decrease of the PV by 10%. PV: Plasma volume.

The rate constant for urine output (*k*_10_) was strongly reduced by sepsis, modestly reduced by β_1_-adrenergic stimulation (Figure 5A), while being increased by α_1_-adrenergic stimulation. Further modifications were minor, but β_1_-adrenergic stimulation accelerated distribution of fluid from the plasma (*k*_12_) and decreased the return flow (*k*_21_), both acting to increase peripheral edema.

A second analysis was performed based on the relationship between hemodynamic variables and kinetic parameters. CO was consistently high in the septic sheep. After controlling the calculations for the effect of sepsis alone, a very high CO was still associated with a lower urine output, higher *k*_12_, and a much greater volume expansion of *V*_t2_ (Supplementary Figure S1). Similar results were obtained using the cardiac index for sheep.^[^^17^^]^

### The pre-infusion PV change

Hemoconcentration indicated that the PV changed by –10% (range +14 to –33) during the 50 min that passed between the administration of lipopolysaccharide and vasopressor. Greater pre-infusion reductions of the PV were associated with significantly higher values of both *k*_21_ and *k*_23,_ while *k*_10_ became reduced. However, the slope for the correlation between the pre-infusion PV change and *k*_23_ was much steeper than for the PV change and *k*_21_, allocating disproportionality more volume to the “third space” if the crystalloid infusion was preceded by greater reductions of the PV. Changes in PV of less than –10% were even associated with negative values of *k*_21_ (Figure 5B) while *k*_23_ remained positive. Hence, at least statistically, a small reduction of PV would be at risk of stopping the lymphatic flow.

## Discussion

### Main findings

Acute sepsis had a marked effect on the hemodynamics by inducing a hyperkinetic, hypotensive, and vasodilatory state. The drug effects were mostly attributed to accelerated diuresis from α_1_-adrenergic stimulation *vs.* water-conserving properties of β_1_-adrenergic stimulation. Overall, the modifying influence of adrenergic stimulation on hemodynamics (Table 2) and fluid distribution (Figure 4) was clearly weaker in the septic sheep than in the non-septic animals. Therefore, it does not seem possible to normalize the fluid distribution by using adrenergic drugs in septic sheep.

The most important kinetic result of sepsis was that the brisk urine flow in the non-septic sheep was replaced by accumulation of fluid in the slow-exchange interstitial fluid space (*V*_t2_), the degree of which was only modestly influenced by the adrenergic drugs.

The results further suggest that the pre-infusion reductions of the PV in the septic sheep increased accumulation of fluid in the “third fluid space” (*V*_t2_). Interestingly, this reduction also seemed to augment the return of fluid from *V*_t1_ to the plasma (the lymphatic flow), which flow could else become virtually arrested (Figure 2E).

### Adrenergic receptors and fluids

Exogenous vasopressors can modify fluid distribution by affecting the microcirculation. In the current study, α_1_-activation reduced the volume expansion of *V*_t1_. This is consistent with its induction of diuresis and its expected vasoconstriction of arterioles and precapillary vessels that would limit capillary flow. Moreover, a_1_-adrenergic activation increases lymphatic return of interstitial fluid,^[^^18^^,^^19^^]^ which also plays a role in keeping *V*_t1_ small. These changes increase the resistance for fluid movement into *V*_t2_.

Conversely, β_1_-activation in the kidney has a marked effect on reducing urine clearance (low *k*_10_), thus expanding the PV and driving fluid into *V*_t1_. At the microcirculatory level, β_1_-activation is associated with a reduction in precapillary resistance and would increase capillary flow (higher *k*_12_) and fluid translocation into *V*_t1_ and, secondarily, to *V*_t2_.

β_1_-Adrenergic receptors are also associated with inhibition of lymphatic pumping (low *k*_21_) and therefore reduced fluid clearance from the interstitial space,^[^^20^^]^ which might be another contributing factor to the expansion of *V*_t1_. These three effects of β_1_-adrenergic stimulation were all confirmed by the covariance part of our fluid kinetic analysis. However, the adrenergic drugs exerted only weak effects in the septic sheep and, therefore, the drug-associated differences in the expansion of *V*_t2_ were not as great as expected (Figure 4F). In addition, dopamine receptors did not seem to affect the fluid distribution at all.

CO was consistently high in the septic sheep. Our interpretation is that the hyperkinetic circulation was not primarily due to activation of adrenergic receptors but to higher plasma nitric oxide concentrations; these reduce SVR and, via Nox (NADPH oxidases) and ROS (reactive oxygen species), causing acute kidney injury and therefore reduced urine flow.

### The slow-exchange “third space”

The concept of the “third fluid space” (*V*_t2_) was introduced in the 1960s by Shires et al.^[^^21^^]^ but has since then often been considered a methodological artifact.^[^^22^^,^^23^^]^ Volume kinetics was recently able to identify and outline the characteristics of this remote space, which probably corresponds to the gel-phase of the interstitium.^[^^9^^]^ *V*_t1_ occupies a space of similar size as *V*_c,_ while a *V*_t2_ of measurable size can only be detected when there is marked fluid overload, or when “suction pressure” (e.g., highly negative interstitial fluid pressure, P_if_) has developed due to the action of inflammatory molecules. In these two settings, *V*_t2_ becomes supraphysiological, which suggests the absence of free flow. Here, the size of *V*_t2_ (obtained as *V*_c_ × *k*_12_ × *k*_23_ / [*k*_21_ × *k*_32_]) was 55 mL in the control experiments and 93 L in the septic sheep (difference 1700 times). Hence, we believe that the opening of *V*_t2_ in the septic sheep provides evidence of disruption of the interstitial matrix.

Accumulation of fluid in the “third space” may create scattered pools of fluid in the tissues (“pitting edema”)^[^^24^^]^ and markedly prolongs the half-life of the infused fluid in the body, from half an hour to several days.^[^^25^^]^ The slow equilibration with the plasma makes the fluid in *V*_t2_ a poor supporter of the PV, whereby hypovolemia may co-exist with peripheral edema.^[^^6^^]^

### Physiology of the “third space”

Accumulation of fluid in *V*_t2_ seems to operate as an overflow reservoir when the body is overloaded with fluid.^[^^25^^]^ Our detection of fluid rapidly entering a “third space” is likely to be the same event as demonstrated by experimental studies by Guyton et al.^[^^26^^,^^27^^]^ in the 1960s. Rapid infusion of crystalloid fluid results in translocation of fluid to the interstitial space and an increase in interstitial fluid pressure, which is normally slightly sub-atmospheric. Rapid expansion of the interstitial space results in fragmentation of matrix elements and opening of the gel-phase with fluid movement into *V*_t2_.

An alternative mechanism for fluid accumulation in *V*_t2_ is what occurs during acute inflammation, for example, burn injuries. Inflammatory mediators, including cytokines, nitric oxide, and proteases, cause alterations in fibroblast–matrix interactions that normally keep the interstitial space compressed; this decreases the interstitial pressure and expands the interstitial space. This suction pressure can reach a magnitude of −100 cm H_2_O and pull large quantities of fluid out of the capillary compartment. This acute reduction of interstitial pressure increases the fluid movement into *V*_t2_.^[^^28^^,^^29^^]^

### Competition between lymph flow and “third-spacing”

The hemoconcentration occurring before crystalloid fluid in the septic sheep was infused shows that a slow hypovolemic process acted in the background. This was probably due to increased capillary leakage of albumin. The decrease of the PV averaged 10% which must have been transferred to *V*_t1_. This shift should be considered when reviewing the lower panel of Figure 4. We believe that the resulting greater volume expansion of *V*_t1_ facilitated early opening of *V*_t2_.

This albumin leakage seemed to prevent the lymphatic flow from becoming completely arrested, as suggested by negative values for *k*_21_ in animals with minor hemoconcentration pre-infusion (Figure 5B). Near-zero lymphatic flow has recently been reported in women with preeclampsia^[^^30^^]^ and is probably due to competition between *k*_21_ and *k*_23_. These two rate constants determine how *V*_t1_ is drained, and a high *k*_21_ maintains the PV while *k*_23_ diverts fluid away from the effective circulation. The ratio *k*_21_/*k*_23_ is normally 2–3 in healthy adults^[^^25^^]^ but was only 0.9 (median) in our septic sheep, showing a preference for fluid to become deposited in *V*_t2_. This supports that a sepsis-specific mechanism allocated fluid to *V*_t2_. The same cytokine and nitric oxide that cause matrix degradation also inhibit lymphatic pumping, further contributing to an expanded interstitial volume.^[^^20^^]^

### Clinical importance

The examined drugs did have effects, but not as strong as in the non-septic animals. Our results also increase understanding of why sepsis-induced plasma leakage is not readily compensated by accelerated lymphatic return, as in healthy subjects (“interstitial washdown”).^[^^31^^]^ The comparison between *k*_21_ and *k*_23_ suggests the existence of suction pressure in *V*_t2_ that reduces the lymphatic return. The combination of suction pressure and direct toxic effects of inflammatory mediators on lymphatic pumping results in hypovolemia, peripheral edema, and hypoalbuminemia (because albumin is circulated via the lymph).^[^^7^^]^ Collectively, at the concentrations used in these studies, adrenergic agents could not overcome the structural derangements of the interstitial space and associated fluid distribution into *V*_t2_ (Figure 4F). Hence, vasopressors do not prevent maldistribution of fluid during sepsis, which highlights the need for clinical therapies that target inflammation and affect the interstitial pressure and lymphatic function.

### Limitations

Our report is a retrospective analysis of two studies that are not fully congruent regarding the examined adrenergic drugs, which is why we used the power of the drugs to affect adrenergic receptors in the covariate analysis.^[^^13^^]^ By contrast, the blood sampling protocol was the same in both studies. Another difference is that the non-septic sheep were studied in the awake state, while the septic animals were anesthetized. Some of the reduction of the urine output in the septic sheep could be due to the anesthesia.^[^^32^^]^ Finally, no data on inflammatory mediators were obtained.

## Conclusions

Vasoactive drugs strongly affected the hemodynamics and the urine output in healthy sheep, while their influence on the distribution of infused saline between fluid compartments was modest. The magnitude of all adrenergic influences on fluid distribution was less pronounced in sheep with acute sepsis. In control animals, infused saline was effectively handled by the kidneys and extravascular fluid expanded the fast exchange compartment (*V*_t1_) within the interstitial space. In contrast, sepsis allocated fluid the slow-exchange interstitial “third fluid space” (*V*_t2_) compartment, which leads to hypovolemia with peripheral edema. Analysis of kinetic parameters is consistent with inflammation-induced disruption of the interstitial matrix, increasingly negative interstitial pressure, e.g., a suction pressure, and inhibition of lymphatic pumping that together lead to the filling of the “third space.”

## CRediT authorship contribution statement

**Robert G. Hahn:** Writing – review & editing, Writing – original draft, Visualization, Validation, Software, Resources, Methodology, Formal analysis, Data curation, Conceptualization. **Yuhong Li:** Writing – review & editing, Supervision, Resources, Project administration, Investigation. **Randal O. Dull:** Writing – review & editing, Writing – original draft, Formal analysis.

## Appendix Supplementary materials

Image, application 1

Image, application 2
