# Supplementary material for: Vasoactive drugs and the distribution of crystalloid fluid during acute sepsis
Source: J Intensive Med. 2025 Oct 17;6(1):75–82. doi: 10.1016/j.jointm.2025.08.008 (PMC12925894; doi:10.1016/j.jointm.2025.08.008)
Supplement: Supplementary file 1 [file mmc1.docx]

**Supplementary File 1**

#### **“**Adrenergic drugs and the distribution of crystalloid fluid in non-septic and acutely septic sheep; a secondary kinetic analysis”

**_______________________________________________________________________**

1. Kinetic model
2. Differential equations, correction equation
3. Covariate models
4. Complete parameter equations
5. Figure S1.
6. Program code for the final model

**Kinetic model**


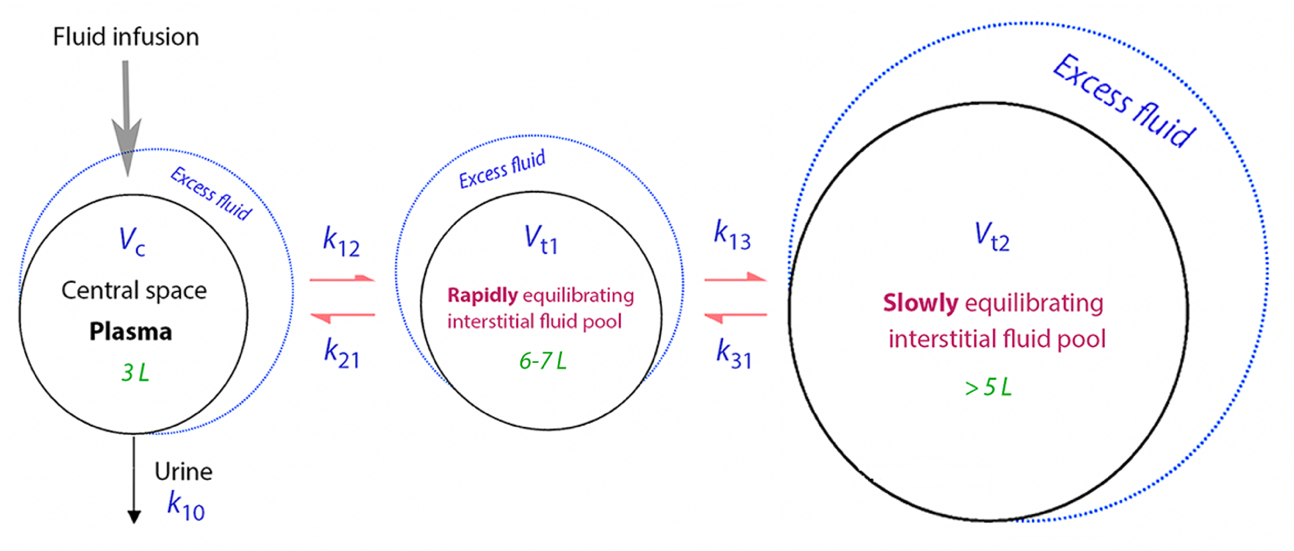


Fluid is infused at the rate *R*_o_ into the plasma, *V*_c_, from which distribution occurs at a rate determined by a rate constant *k*_12_ to a rapid-exchange interstitial space, *V*_t1_. The distributed fluid returns to *V*_c_ at a rate determined by a constant *k*_21_. Fluid is also distributed from *V*_t1_ to a slow-exchange interstitial space, *V*_t2_, at a rate determined by a constant *k*_23_. The return of this fluid to *V*_t1_ is governed by a rate constant *k*_32_. Urinary excretion (U) occurs in proportion to the expansion of *V*_c_ by a rate constant *k*_10_. Hence, the two interstitial compartments are connected to *V*_c_ in a serial fashion.

The differential equations are:

 d*v*_c_ /dt = *R*_o_ – *k*_12_ (*v*_c_ – *V*_c_) + *k*_21_ (*v*_t1_ – *V*_t1_) – *k*_10_ (*v*_c_ – *V*_c_)

d*v*_t1_ /dt = *k*_12_ (*v*_c_ – *V*_c_) – *k*_21_ (*v*_t1_ – *V*_t1_) – *k*_23_ (*v*_t1_ – *V*_t1_) + *k*_32_ (*v*_t2_ – *V*_t2_)

d*v*_t2_ /dt = *k*_23_ (*v*_t1_ – *V*_t1_) – *k*_32_ (*v*_t2_ – *V*_t2_)

dU /dt = *k*_10_ (*v*_c_ – *V*_c_)

Expanded volumes are indicated by lower-case letters (*v*_c_, *v*_t1,_ and *v*_t2_) and baseline volumes by capital letters (*V*_c_, *V*_t1,_ and *V*_t2_). The hemodilution [(Hb/hb)–1] was divided by (1– baseline hematocrit) to obtain the Hb-derived fractional plasma dilution, which corresponds to (*v*_c_ – *V*_c_) / *V*_c_ and increases in a zero-order linear fashion with additions of fluid to the kinetic system. The excreted urine, whenever collected, is used as input variable for *U*. The glycocalyx volume is part of *V*_c_ [Intensive Care Med Exp 2020; 8: 29].

**Correction equation**

Each calculation of dilution underwent a minimal correction to account for blood sampling.

The calculations were performed as follows: the initial blood volume (BV_0_) was first obtained using Nadler’s formula, where the total blood volume prior to the infusion of 20% albumin (BV_0_) was derived from the height (*h*) in meters, body weight (*w*) in kilograms, and sex (Surgery 1962; 51: 224-232)

Male: BV_0_ = 0.3669 *h*^3^ + 0.03219 *w* + 0.6041

Female: BV_0_ = 0.3561 *h*^3^ + 0.03308 *w* + 0.1833

The blood volume at a later time 1 was calculated by first estimating the total hemoglobin mass in the circulation at baseline (Hb_mass0_) as being equal to the product of BV_0_ and the blood Hb concentration at baseline, Hb_0_. Losses from Hb_mass_ were then subtracted were then subtracted for each measurement, and BV_1_ obtained by dividing this difference by a freshly taken Hb. PV = plasma volume: Hence:

Hb_mass_ _0_ = BV_0_ Hb_0_

Hb_mass_ _1_ = Hb_mass_ _0_ – (blood loss _0-1_ (Hb_1_ + Hb_o_) / 2)

BV_1_ = Hb_mass_ _1_ / Hb_1_

PV_1_ = BV_1_ (1 – hematocrit_0_ Hb_1_ / Hb_0_ )

(*v*_c_ – *V*_c_) = (PV_1_ – PV_0_) / PV_0_

were PV is the plasma volume. The principle is to use to use Hb as a biomarker of dilution while sequentially correcting the intravascular hemoglobin mass for losses due to blood sampling and bleeding. If this is not done, the plasma dilution values will be falsely high.

This correction can be overlooked no surgical hemorrhage occurs and only small volumes of blood are sampled. The plasma dilution can then be obtained directly:

[(Hb_0_ / Hb_1_) – 1] / ( 1– hematocrit_0_ )

**Covariate models**

The rate constants and *V*_c_ could all be modified by *covariates*, which are characteristics that may change the parameter estimates in a specific individual or in a sub-group. The most promising candidates for covariate effects was searched by plots of random effects ("eta:s"). These variables were then added one by one to the model and accepted if the -2 log likelihood (-2 LL) for the model then decreased by > 3.84 points, which corresponds to *P*< 0.05. A decrease by > 6.6 points represents a significance level of *P*< 0.01.

Several covariates used the *exponential model* which is appropriate for categorical variables. For example, the rate parameter *k*_10_ had the group value of 0.0474 min^-1^ but associated with a covariate effect of -2.94 in the septic pigs. Thus, the following modified value of *k*_10_ should be used for the septic pigs:

*k*_10_ = 0.0474 [e ^–2.94^] = 0.0025

as e = 2.718. Thus, the rate constant for the dilution-dependent diuretic response to plasma volume expansion (*k*_10_) is only 5% of the value that is valid for the non-septic sheep.

Continuous data that include 0 or negative values are analyzed according to the *linear covariate model.* An example is the covariance between β_1_-receptor stimulation and *k*_21_. Here, the group value was 0.0635 min^-1,^ the covariance effect -0.40, and the mean for all experiments 0.62. The value for *k*_21_ in a patient receiving a vasopressor with the score of 2 (see **Table 1**) the becomes

*k*_21_ = 0.0635 [1 - 0.40 (2 – 0.62)] = 0.0284

Thus, the rate constant *k*_21_ is decreased to 45% of the mean value for all experiments in response β_1_-receptor stimulation of score 2 in intensity accoriding to the scale shown in **Table 1.**

One parameter could be subject to several covariate effects. In the present study, this occurred for *k*_10_ and *k*_23_, as shown in **Table 1** and below**.**

**Complete parameter equations**

The compete equations for all parameters shown in Table 3 of the main manuscript are:

*V*_c_ = 1220 mL

*k*_12_ = 0.0567 [(1 + 0.0628 (β_1_ score – 0.62 ) ]

*k*_21_ = 0.0635 [(1 - 0.16 (β_1_ score – 0.62)]

*k*_23_ = 0.0000447 [e ^7.43 (non-sepsis=0, sepsis = 1)^ ] [e ^-6.40 (after infusion=0, during infusion = 1)^ ]

*k*_32_ = 0.000869

*k*_10_ = 0.0474 (e ^-2.94 (non-sepsis=0, sepsis = 1)^ ] [(1 – 0.40 (β_1_ score – 0.62)] [(1 + 0.45 (α1 score – 0.94)]


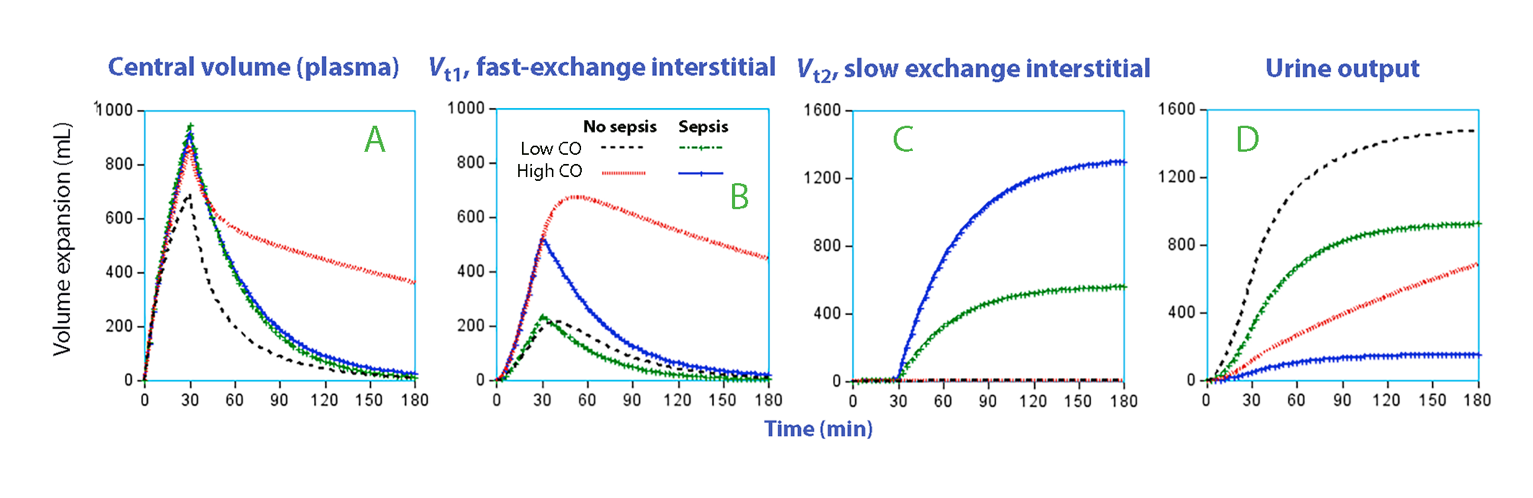


**Fig. S1**

Simulation of the distribution of 1.5 L of isotonic saline infused over 30 min in sheep with and without sepsis depending on cardiac output being 3 L/min (“Low”) or 15 L/min (“High”), irrespective of vasoactive therapy. Plots are controlled for the independent effect of MAP on urine output (*k*_10_) as well as of sepsis on the rate of entrance of fluid to *V*_t2_ (*k*_23_).

**Program code for the final model**

test(){

deriv(A1 = - (A1 * Ke)- (A1 * K12- A2 * K21))

urinecpt(A0 = (A1 * Ke))

deriv(A2 = (A1 * K12- A2 * K21)- (A2 * k23- A3 * k32))

deriv(A3 = (A2 * k23- A3 * k32))

C = A1 / V

dosepoint(A1, idosevar = A1Dose, infdosevar = A1InfDose, infratevar = A1InfRate)

error(CEps = 0.0399603129388893)

observe(CObs = C + CEps)

error(A0Eps = 283.10271487088)

observe(A0Obs = A0 + A0Eps)

stparm(V = tvV * exp(nV))

stparm(Ke = tvKe * (1+(Beta1-mean(Beta1))*dKedBeta1) * (1+(Alpha1-mean(Alpha1))*dKedAlpha1) * exp(dKedSepsis1*(Sepsis==1)) * exp(nKe))

stparm(K12 = tvK12 * (1+(Beta1-mean(Beta1))*dK12dBeta1) * exp(nK12))

stparm(K21 = tvK21 * (1+(Beta1-mean(Beta1))*dK21dBeta1) * exp(nK21))

stparm(k23 = tvk23 * exp(dk23dSepsis1*(Sepsis==1)) * exp(dk23dUnderefter1*(Underefter==1)) * exp(nk23))

stparm(k32 = tvk32 * exp(nk32))

fcovariate(BW)

fcovariate(MAP)

fcovariate(CO)

fcovariate(CVP)

fcovariate(Alpha1)

fcovariate(Beta1)

fcovariate(Dopamin)

fcovariate(Sepsis())

fcovariate(Underefter())

fixef(tvV = c(, 1265.11428372396, ))

fixef(tvKe = c(, 0.06302531079567, ))

fixef(tvK12 = c(, 0.0545088173842112, ))

fixef(tvK21 = c(, 0.0610842083929338, ))

fixef(tvk23 = c(, 4.14079559005819E-05, ))

fixef(tvk32 = c(, 0.00027898457804958, ))

fixef(dKedSepsis1(enable=c(0)) = c(, -3.31132799715979, ))

fixef(dk23dSepsis1(enable=c(1)) = c(, 7.33622559741474, ))

fixef(dK12dBeta1(enable=c(2)) = c(, 0.136693604730742, ))

fixef(dK21dBeta1(enable=c(3)) = c(, -0.111731950684525, ))

fixef(dKedBeta1(enable=c(4)) = c(, -0.398855439864739, ))

fixef(dKedAlpha1(enable=c(5)) = c(, 0.610569936191777, ))

fixef(dk23dUnderefter1(enable=c(6)) = c(, -3.43870446075765, ))

ranef(block(nKe, nK12, nK21, nV, nk23, nk32) = c(0.94989369, 0, 0.4113892, 0, 0, 0.10833959, 0, 0, 0, 0.068108715, 0, 0, 0, 0, 2.0049258, 0, 0, 0, 0, 0, 72.041592))

}
